# A viscoelastic-plastic model for the core of various close-packings of multifilament polyamide-6 yarns

**DOI:** 10.1038/s41598-024-74602-2

**Published:** 2024-10-11

**Authors:** Milad Razbin, Mortaza Salehian, Ali Akbar Gharehaghaji

**Affiliations:** 1 Department of Textile Engineering, Amirkabir University of Technology, Tehran, Iran; 2 Department of Aerospace Engineering, Amirkabir University of Technology, Tehran, Iran

**Keywords:** Multi-filament yarn, Tensile properties, Visco-elasto-plastic behavior, Geometrical modeling, Artificial neural network, Mechanical engineering, Mechanical properties, Computational science

## Abstract

Different forms of close-packed yarns can be produced by varying the number of monofilaments in the core region, ranging from one to five. Numerous efforts have been made to model or simulate the mechanical response of close-packed yarns; however, previous studies have predominantly focused on one or two monofilaments in the core. In this study, we propose an analytical approach that combines a geometrical model with an artificial neural network (ANN) to predict the tensile behavior of close-packed yarns containing 2 to 5 monofilaments in the core region. The novelty of this hybrid model lies not only in accounting for more than two monofilaments in the core but also in extending the prediction range from elastic to viscoelastic-plastic behavior. Validation of the proposed method showed excellent agreement between experimental and theoretical results. Numerical simulations further confirmed that the results align with theoretical predictions, demonstrating the model’s accuracy in predicting the tensile behavior of close-packed yarns. This modeling approach has the potential to significantly improve the understanding and modeling of textile structures.

## Introduction

The mechanical properties of yarns play an important role in determining the mechanical properties of fabrics such as woven, knitted, and braided yarns^1^. Yarns can be categorized into two main types: spun and filament. Spun yarns are produced from staple fibers using processes such as ring spinning, whereas filament yarns are made from continuous monofilaments through processes like melt spinning. In staple fibers, a high level of twisting increases the friction between fibers, thereby enhancing performance under tension. In continuous fibers, a low level of twisting prevents separation of fibers^1,2^. Modeling concepts for textiles have been introduced in a manner similar to those used for materials such as wood, steel, and concrete. Several attempts have been made to develop models that predict the tensile behavior of yarns^3–5^. Various methods, including the force method, energy method, numerical simulation, and experimental modeling, have been employed for prediction of tensile properties.

Regarding force methods, multiple formulas have been derived to examine how geometrical yarn parameters impact tensile behavior under cyclic loading for both continuous and staple fiber yarns^6^. It has been shown that a reduction in Young’s modulus of filamentous yarns occurs due to increased twist, which causes uniform stress distribution^7^. Kruse and Soliman presented a model that predicted the strength of false-twist yarns using ideal yarn structure variables such as displacement of twisted fiber, twist angle, strain, friction, and slenderness^8^. Grabowska developed a model predicting the tensile loading of multi-ply loop yarns based on variables such as fiber length distribution, migration, slip, twist, tensions, and friction^9^. Huang et al.^10^ established a model predicting the strength of two-ply yarns by considering axial forces and inter-filament friction. However, experimental deviations occurred due to the assumptions made in the model. Zubair et al. developed an analytical model for staple fiber cotton by considering the stress-strain curve, twist angle, fiber distribution, and contraction ratio to predict the yarn stress-strain curve^10^.

Using the energy method, Hearle et al. first proposed the “ideal yarn” model, conceptualizing yarns as concentric cylinders with filaments arranged in helical paths, capable of representing real yarns^11^. Postle et al. derived yarn torque as the sum of fiber tension, torsion, and bending, concluding that fiber torsion significantly impacts yarn structure. Pan studied the effects of slippage and orientation on the mechanics of staple yarns^12^. Tandon et al. modeled the torsion of single-ply yarns while maintaining a constant length^13^. Jiang et al. proposed the “shortest path” theory using discrete fiber modeling and an energy approach, introducing a changing-pitch system^14^. In another reported work, Liu et al. suggested a mechanical model for wool yarns based on discrete fiber modeling and determined the fiber paths by minimizing strain energy^15^. Du et al. demonstrated that yarn cross-sections should be elliptical under compression. By considering bending and torsional energies, they formulated the rigidity of filament yarns^16^.

In numerical simulations, He et al.^17^ modeled staple fiber yarns with diameter irregularities using the finite element method (FEM). Irregularities with higher magnitudes and frequencies decreased properties such as strength, elongation, and elastic modulus. They reported that increasing the gauge length did not impact strength unless weak spots appeared. In further work, they modeled the non-linear behavior of staple fibers with irregularities^18^. Sreprateep and Bohez presented an algorithm to model multi-ply yarns based on “virtual location,” capable of representing both ideal and real yarn properties^17^. Sreprateep and Pattiya compared parametric equations, splines, and non-uniform rational B-splines (NURBS) to approximate fiber paths. Splines and NURBS provided smoother fiber migration curves, while parametric equations were better suited for ideal yarns^19^. Striprateep and Bohez presented a computer-aided design/computer-aided engineering (CAD/CAE) method to predict the tensile behavior of multi-filament yarns^20^.

In terms of experimental modeling, Ramesh et al. and Langenhove and Sette discussed the relationship between air-jet spinning machine input variables (yarn count, blend, nozzle pressures) and output variables such as breaking load and elongation using feed-forward back-propagation networks^21–23^. Majumdar et al. utilized adaptive neuro-fuzzy systems and feed-forward back-propagation networks to predict the properties of ring and rotor spun cotton yarns^24,25^. Ureyen and Gurkan used a feed-forward back-propagation network model to predict the tenacity and elongation of ring spun yarns based on count, twist, and roving properties^26,27^. Almetwally et al. employed a feed-forward back-propagation network to predict the properties of core-spun yarns^28^. For predicting the intermingled yarn properties, Ozkan et al.^29^ compared feed-forward back-propagation and linear regression models. Ghanmi et al. used response surface methodology and neuro-fuzzy neural networks to predict the strength and elongation of ring spun yarns based on fiber and spinning parameters^30,31^. In an attempt for prediction of the tenacity and elongation of rotor spun yarns, Malik et al.^32^. adopted a feed-forward back-propagation network. Yildirim et al. investigated nonlinear regression and feed-forward back-propagation using BFGS training to predict the properties of polyester partially oriented yarn (PET-POY)^29^. A grey wolf optimizer-based feed-forward back-propagation network was coupled with response surface methodology by Hadavandi et al. to predict the strength of Siro-spun yarns^33^.

In addition to the aforementioned methods, Rao and Farris used an orthotropic composite theory model to demonstrate the impact of twist level on the yarn modulus^34^. Pan et al. proposed a simple model based on fiber length and diameter variations, twist level, and packing density to predict cotton yarn strength^32^. Cartraud and Messager^35^ used homogenization theory to determine stiffness coefficients for 7-ply structures. They found that homogenization is useful for estimating the mechanical properties of 7-ply yarns. Yang et al. developed a viscoelastic model incorporating Kelvin, Maxwell, and spring elements to predict the non-linear stress-strain behavior of co-wrapped composite yarns^36^. Recently, Razbin et al. used an elasticity approach to model the longitudinal Young’s modulus of helical auxetic yarn-reinforced composites^37^.

Based on literatures, it can be said that analytical models are restricted to linear regions due to simplifying assumptions. Soft computing methods rely on experimental data which is time-consuming. Computer-aided methods have limitations due to simplifications and fail to provide equations that can be applied in practical use. Most yarns are made of polymeric fibers, with mechanical properties dependent on strain, strain rate, temperature and humidity, which all making a comprehensive model complex. Hence, a general superior and innovative modeling method is necessary. In our previous work^38^, we demonstrated that integrating a feed-forward back-propagation network into a geometrical model to predict the tensile behavior of open-packed yarns resulted in a highly accurate hybrid model. In the current study, we focus on the core region of close-packed yarns, applying the same approach to showcase the efficiency of this combination in terms of modeling. The proposed viscoelastic-plastic model is not restricted to linear regions. The model inputs are strain, strain rate, twist level, diameter, Poisson’s ratio, and number of monofilament. An artificial neural network maps monofilament strain and strain rate to force, modeling monofilament tensile behavior. The model is verified experimentally at 4 number of cores, 3 twist levels and 2 strain rates. The performance of the proposed model is investigated based on comparing experimental and simulated data. In summary, the proposed viscoelastic-plastic model aims to address limitations of previous approaches by considering number of monofilament in core region, twisting effects and rate of strain.

## Modeling

### Geometrical analysis

#### Basis assumptions

For modeling, several simplifying assumptions have been adopted. It is assumed that all monofilaments are positioned identically (resulting in the same strain under tensile loading) and have circular cross-sections that remain unchanged throughout the tensile loading process. Each monofilament is considered a homogeneous material, and frictional forces between the components are neglected. The Poisson’s ratio of the monofilaments is assumed to remain constant during tensile loading, and no torsional effects are present, meaning the monofilaments equally share the applied load.

#### Geometrical parameters before and after the loading condition

**Figure 1:**
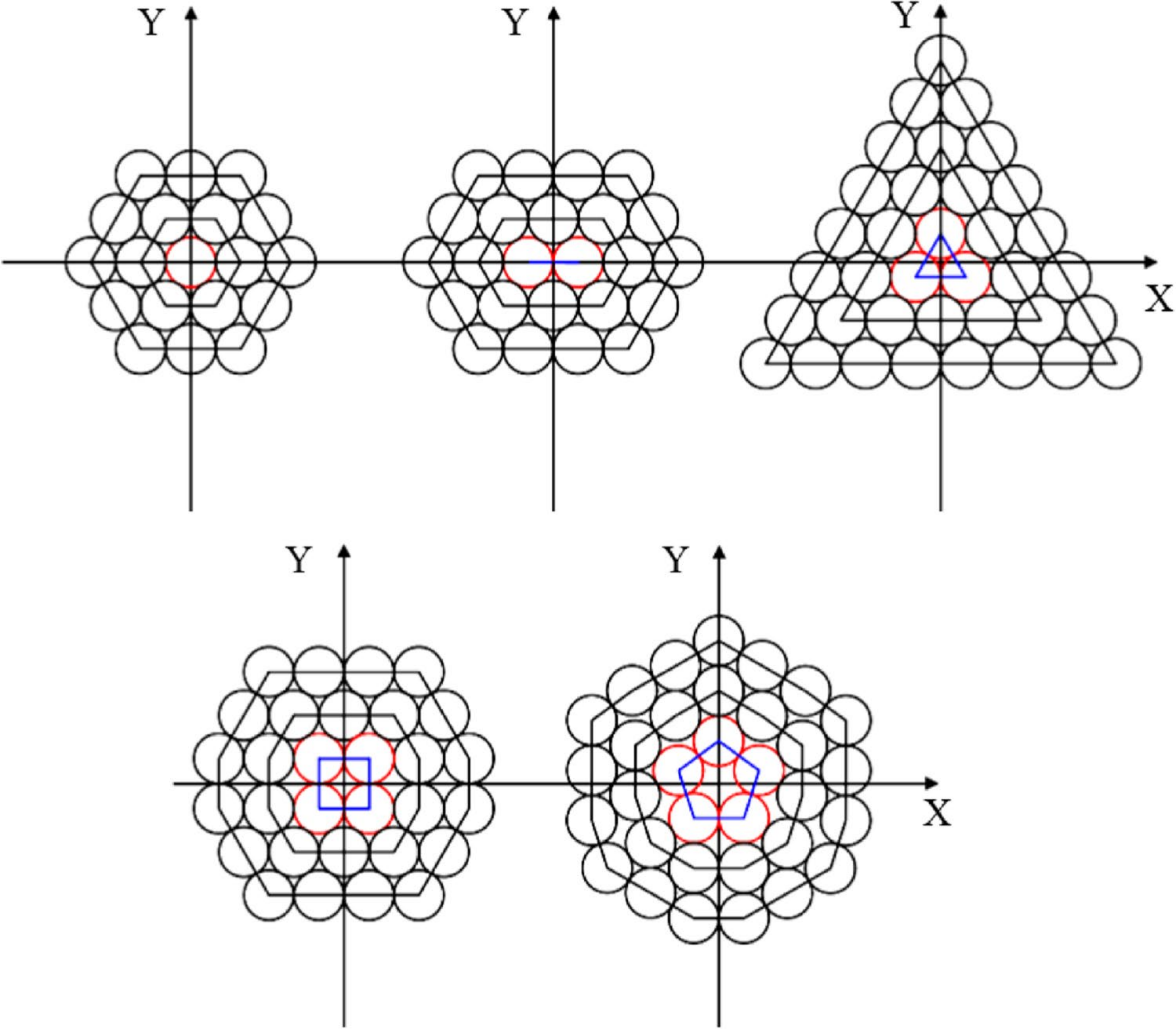
Different form of close-packing yarns.

Generally, the structure of plied yarn can be categorized into two forms including open-packing and close-packing. In open-packing, the monofilaments are arranged in layers between successive concentric circles. In close-packing, the monofilaments fit into polygonal patterns, with the number of sides depending on the number of core fibers formed during the spinning process^1^. Figure 1 illustrates cross-sections of close-packing yarn based on the number of cores.

**Figure 2:**
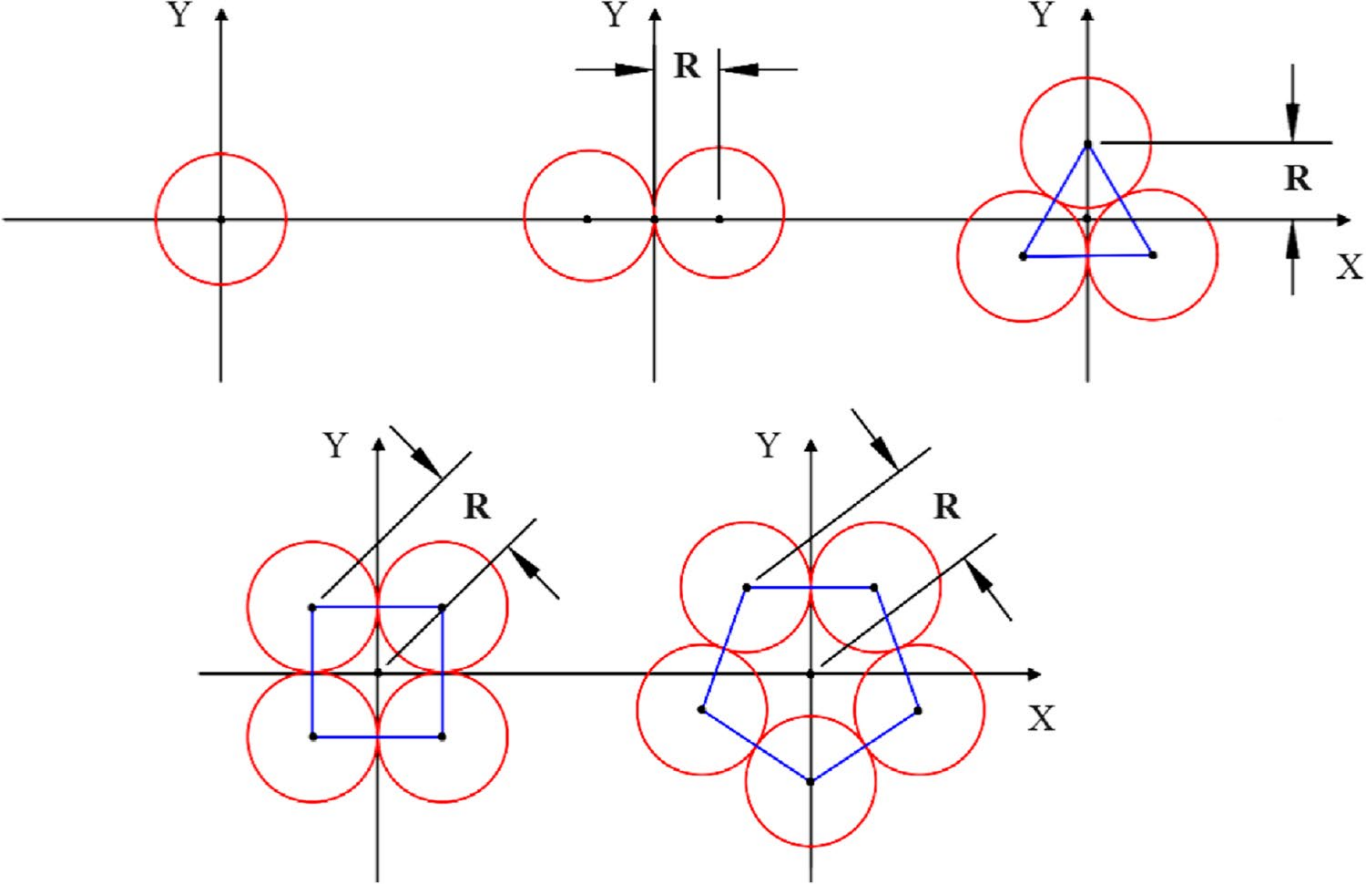
Cross-section of core of close-packing yarns formed by one, two, three, four, and five monofilaments.

This study aims to propose a model to predict the tensile behavior of sections where core monofilaments are situated. Figure 2 depicts the layer radii of yarns formed using two, three, four, and five monofilaments.

Accordingly, the distance between the center of the yarn structure and the center of the monofilaments can be expressed using Eq. (1).

1 $$\:{R}_{0}={r}_{0}{csc}\left(\frac{\pi\:}{N}\right)$$

**Figure 3:**
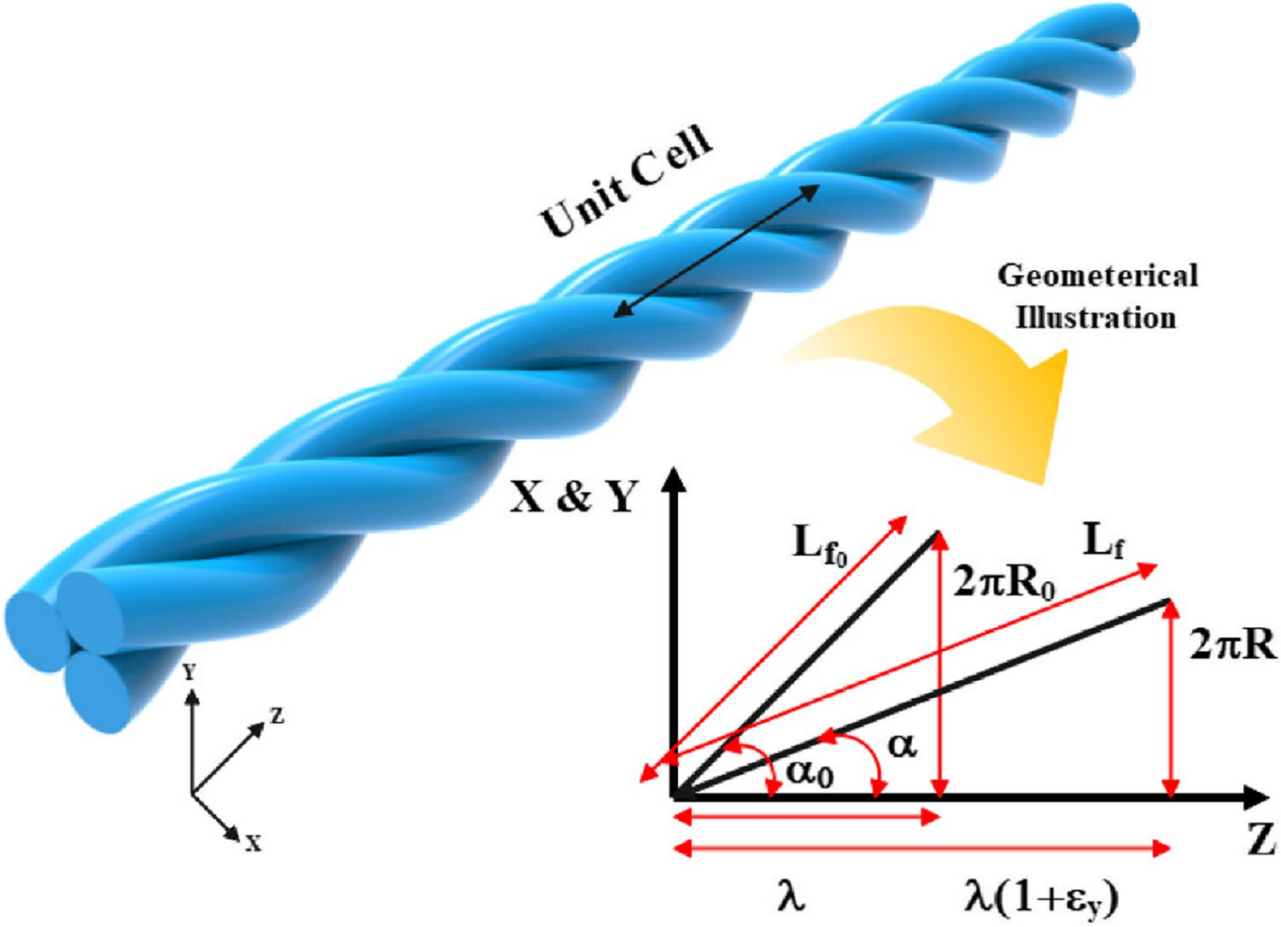
Longitudinal view of the structures before and after the tensile loading condition.

Where $\:R$, $\:r$, and $\:N$ represent the layer radius, monofilament radius, and the number of monofilament, respectively. The index 0 indicates the initial state of the structure. Consequently, the geometrical parameters of a complete twist of the structure, viewed longitudinally as a unit cell, are illustrated in Fig. 3.

Based on Fig. 3, the pitch length ($\:\lambda\:$), the length of monofilament per pitch ($\:{L}_{f}$), the twisting angle ($\:\alpha\:$), and the cross-sectional area of the yarn ($\:{A}_{y}$​) can be formulated using the following Equations^1^:

2 $$\:\lambda\:=\frac{1}{{TPM}_{0}}$$

3 $$\:{{L}_{f}}_{0}=\sqrt{{\lambda\:}^{2}+{\left(2\pi\:{R}_{0}\right)}^{2}}=\sqrt{{\lambda\:}^{2}+4{\pi\:}^{2}{{r}_{0}}^{2}{{csc}}^{2}\left(\frac{\pi\:}{N}\right)}$$

4 $$\:{\alpha\:}_{0}={{tan}}^{-1}\left(\frac{2\pi\:{R}_{0}}{\lambda\:}\right)={{tan}}^{-1}\left(\frac{2\pi\:{r}_{0}{csc}\left(\frac{\pi\:}{N}\right)}{\lambda\:}\right)$$

5 $$\:{{A}_{y}}_{0}=N\pi\:{{r}_{0}}^{2}\mathrm{s}\mathrm{e}\mathrm{c}\left({\alpha\:}_{0}\right)$$

When the structure is subjected to tensile loading, the length of the monofilament will change. Equation (6) expresses the new length of the monofilament based on the changes in pitch length and layer radius:

6 $$\:{L}_{f}=\sqrt{{\left(\lambda\:\left(1+{\epsilon\:}_{y}\right)\right)}^{2}+{\left(2\pi\:R\right)}^{2}}$$

Conversely, the new layer radius can be calculated based on the new radius of the monofilament.

7 $$\:R=r{csc}\left(\frac{\pi\:}{N}\right)$$

Considering Poisson’s effect and the definition of engineering strain, we can derive the following relationship^39^:

8 $$\:r={r}_{0}(1-{\nu\:}_{f}{\epsilon\:}_{f})$$

9 $$\:{\epsilon\:}_{f}=\frac{{L}_{f}-{{L}_{f}}_{0}}{{{L}_{f}}_{0}}$$

Where $\:{\nu\:}_{f}$ refers to the Poisson’s ratio of the monofilament. By substituting Eqs. (7), (8), and (9) into Eq. (6), we obtain:

10 $$\:{L}_{f}=\sqrt{{\left(\lambda\:\left(1+{\epsilon\:}_{y}\right)\right)}^{2}+4{\pi\:}^{2}{{r}_{0}}^{2}{{csc}}^{2}\left(\frac{\pi\:}{N}\right){\left(1-{\nu\:}_{f}\frac{{L}_{f}-{{L}_{f}}_{0}}{{{L}_{f}}_{0}}\right)}^{2}}$$

Equation (10) is a second-order equation with a deterministic solution. By solving it, the new twisting angle of the monofilament can be estimated using Eq. (11).

11 $$\:\alpha\:={{tan}}^{-1}\left(\frac{2\pi\:R}{\lambda\:(1+{\epsilon\:}_{y})}\right)={{tan}}^{-1}\left(\frac{2\pi\:r{csc}\left(\frac{\pi\:}{N}\right)}{\lambda\:(1+{\epsilon\:}_{y})}\right)$$

In this section, the structural parameters after subjection to loading condition, including the pitch length, the length of the monofilament per pitch, and the twisting angle under tensile loading conditions, are formulated. The next section is focused on mechanical analysis to determine the stress-strain curve for core of close-packing yarns.

### Mechanical analysis

#### Total stress acting on the structure

**Figure 4:**
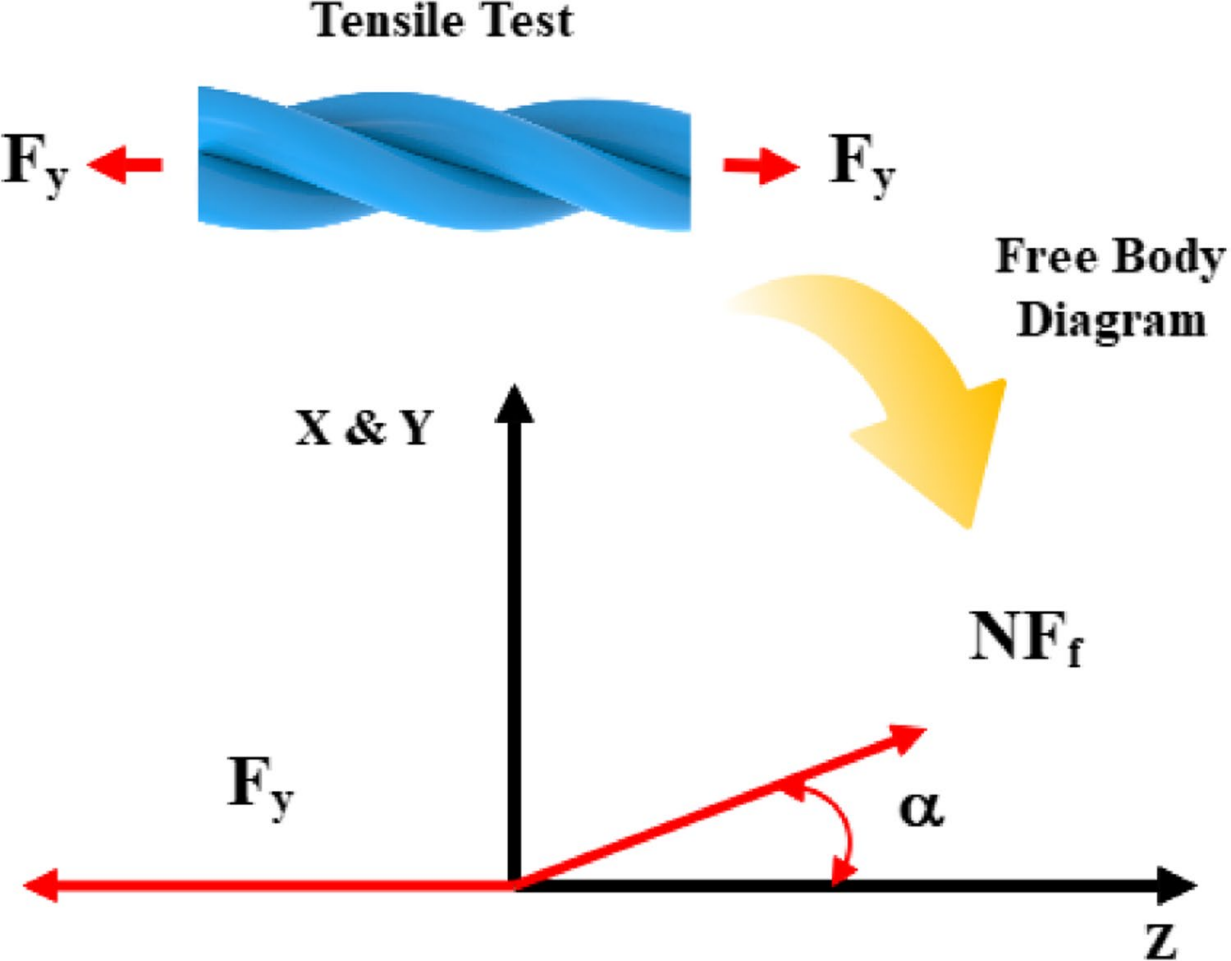
Free body diagram of the structure under tensile loading conditions.

Figure 4 represents the free body diagram of the structure under the loading condition. According to this figure, the force acting on each monofilament is represented as the horizontal component of the total force acting on the structure, divided by the number of monofilaments.

By taking into account the number of monofilaments, the force acting on a single monofilament, and the twisting angle, the total force acting on the structure can be expressed as Eq. (12)^35^.

12 $$\:{F}_{y}=N{F}_{f}\mathrm{s}\mathrm{e}\mathrm{c}\left(\alpha\:\right)$$

To convert the force into engineering stress, we can write^39^:

13 $$\:{\sigma\:}_{y}=\frac{{F}_{y}}{{{A}_{y}}_{0}}=\frac{{F}_{f}\mathrm{s}\mathrm{e}\mathrm{c}\left(\alpha\:\right)}{\pi\:{{r}_{0}}^{2}\mathrm{s}\mathrm{e}\mathrm{c}\left({\alpha\:}_{0}\right)}$$

It is shown that the total force acting on the structure depends on the number of monofilaments, the force acting on each single monofilament, and the twisting angle. The twisting angle can be estimated using geometrical formulas. However, the force acting on a single monofilament depends on the amount of applied strain, which can follow a linear relationship as described by Hooke’s law. In this work, we will utilize an artificial neural network (ANN) method to construct a non-linear relationship that encompasses the visco-elasto-plastic behavior of the monofilament. The details of this approach will be explained in the next section.

#### Determination of tensile function via artificial neural network

Generally, there is no universal method to express the tensile behavior of materials under different loading conditions. Typical methods often simplify material behavior into linear models, which can limit the application of proposed analytical models. Additionally, some non-linear methods rely on specific independent parameters. In contrast, models based on ANN offer a versatile approach capable of mapping any number of independent parameters to a particular dependent parameter^40,41^. ANNs have demonstrated high accuracy and precision in predictions; however, their performance depends on the accuracy and precision of the experimental data^42,43^. It has been demonstrated that integrating an ANN with either analytical^38^or numerical^44,45^ methods enhances the prediction accuracy and capability. Thus, in this study, a feed-forward back-propagation network was employed to establish a function that maps applied strain and strain rate to the corresponding force in polyamide-6 (PA-6) monofilaments. A data split ratio of 90:10 was applied, resulting in 1,059 data points being used for training and 117 for testing the network. The network architecture consists of 2 neurons in the input layer, 8 neurons in the hidden layer, and 1 neuron in the output layer. The number of neurons in the hidden layer was determined using the trial-and-error method. It has been demonstrated that using a tansigmoid transfer function for the hidden layer and a pure linear transfer function for the output layer improves the network’s prediction capability^46,47^. Therefore, the same functions were employed in this study. Both the learning rate and momentum were set to 0.9. The Levenberg-Marquardt algorithm was employed as the training function, with the number of epochs limited to 1,000. Mathematically, the input and output of the developed ANN model can be expressed as follows:

14 $$\:{F}_{f}=Net({\epsilon\:}_{f},{\dot{\epsilon\:}}_{f})$$

MATLAB software was used to construct such a network and the settings are outlined in Table 1.

**Table 1:** Hyperparameter value of the artificial neural network model.

Parameter	Value
Data split ratio (train: test)	90:10
Number of neurons in input layer	2
Number of neurons in hidden layer	8
Number of neurons in output layer	1
Activation function of hidden layer	Tangent sigmoid
Activation function of output layer	Pure linear
Learning rate	0.90
Momentum value	0.90
Learning function	Levenberg-Marquardt
Number of training cycles (epochs)	1000

It is important to mention that the values of the parameters were normalized between 0.1 and 0.9 before being fed into the network to avoid any quantitative effects. This normalization was performed using Eq. (15).

15 $$\:{x}_{n}=0.8\left(\frac{{x}_{a}-{x}_{min}}{{x}_{max}-{x}_{min}}\right)+0.1$$

**Figure 5:**
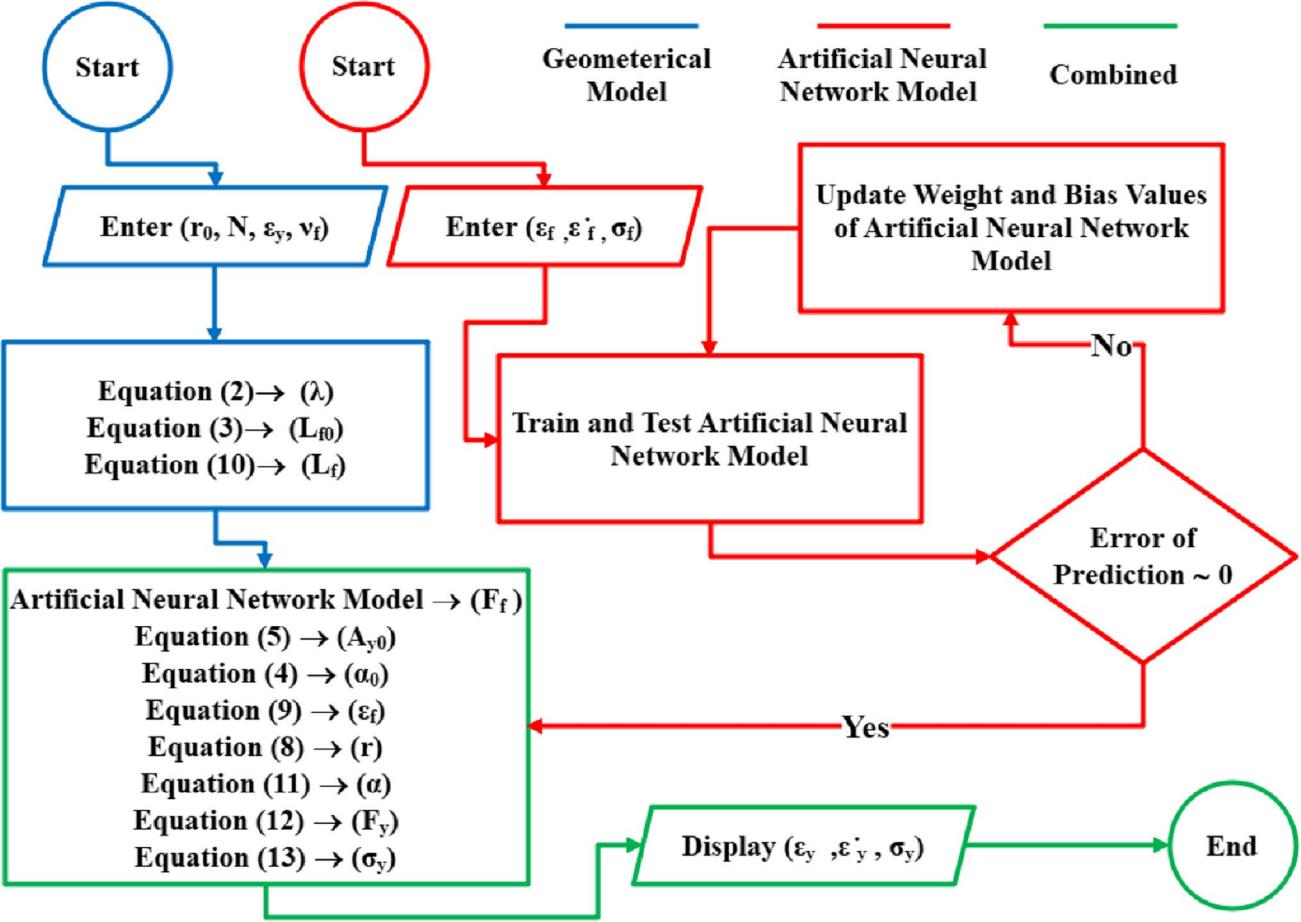
Flowchart of proposed viscoelastic-plastic model to predict tensile behavior of plied yarns.

Where $\:{x}_{a}$, $\:{x}_{max}$, and $\:{x}_{min}$​ are the actual, maximum, and minimum values of the parameter, respectively. To provide an insight into the proposed model and highlight the contribution of this study within the textile field, Fig. 5 presents a flowchart of the viscoelastic-plastic model designed to predict the tensile behavior of plied yarns. The developed model integrates a geometrical model to calculate the length of the monofilament at varying yarn strains, alongside an ANN model to estimate the corresponding force applied to the monofilament. Subsequently, the total force acting on the yarn is determined using the force method, enabling the prediction of the yarn’s stress-strain behavior.

## Experimental section

### Materials and experimental design

In this study, Polyamide-6 (PA-6) monofilament with a diameter of 0.50 mm was purchased from Bushehr Nylone Nakh Company (Tehran, Iran) to fabricate the yarns. An experimental design was implemented to investigate the effects of strain rate, the number of monofilaments, and twists per meter on the tensile behavior of the yarns on various specimens as outlined in Table 2..

**Table 2:** Experimental design.

Sample	No. core	Twist per meter (cm^-1^)	Rate of strain (min^-1^)
1	1	-	1.2
2	2	100	1.2
3	3	100	1.2
4	4	100	1.2
5	5	100	1.2
6	3	200	1.2
7	3	300	1.2
8	3	100	0.1
**Symbol**	**Description**	**Symbol**	**Description**
R	Layer radius	$\nu$	Poisson’s ratio
r	Monofilament radius	F	Force
N	Number of monofilaments	$\sigma$	Stress
$\lambda$	Pitch length	$\dot\varepsilon$	Strain rate
TPM	Twist per meter	ANN	Artificial neural network
L	Length	**Index**	**Description**
$\alpha$	Twisting angle	0	First stage
A	Cross-sectional area	f	monofilament
$\varepsilon$	strain	y	Multifilament yarn

### Fabrication and characterization methodologies

To produce the yarns, eight samples were prepared using a twisting device. During the manufacturing process, the monofilaments were mounted between the clamps in parallel and twist was inserted. Additionally, before applying the twist, the left-side clamp was allowed to move to compensate the reduction in length.

Tensile tester (Instron 5566) was used to conduct the tensile tests on monofilaments and yarns according to ASTM D 3822-01. These tests were conducted under various strain rates, with a gauge length of 250 mm, at a temperature of 22 °C, and a humidity of 44%. Each test was performed five times on each yarn sample until its rupture.

## Results and discussion

### Correlation between experimental and theoretical values

**Figure 6:**
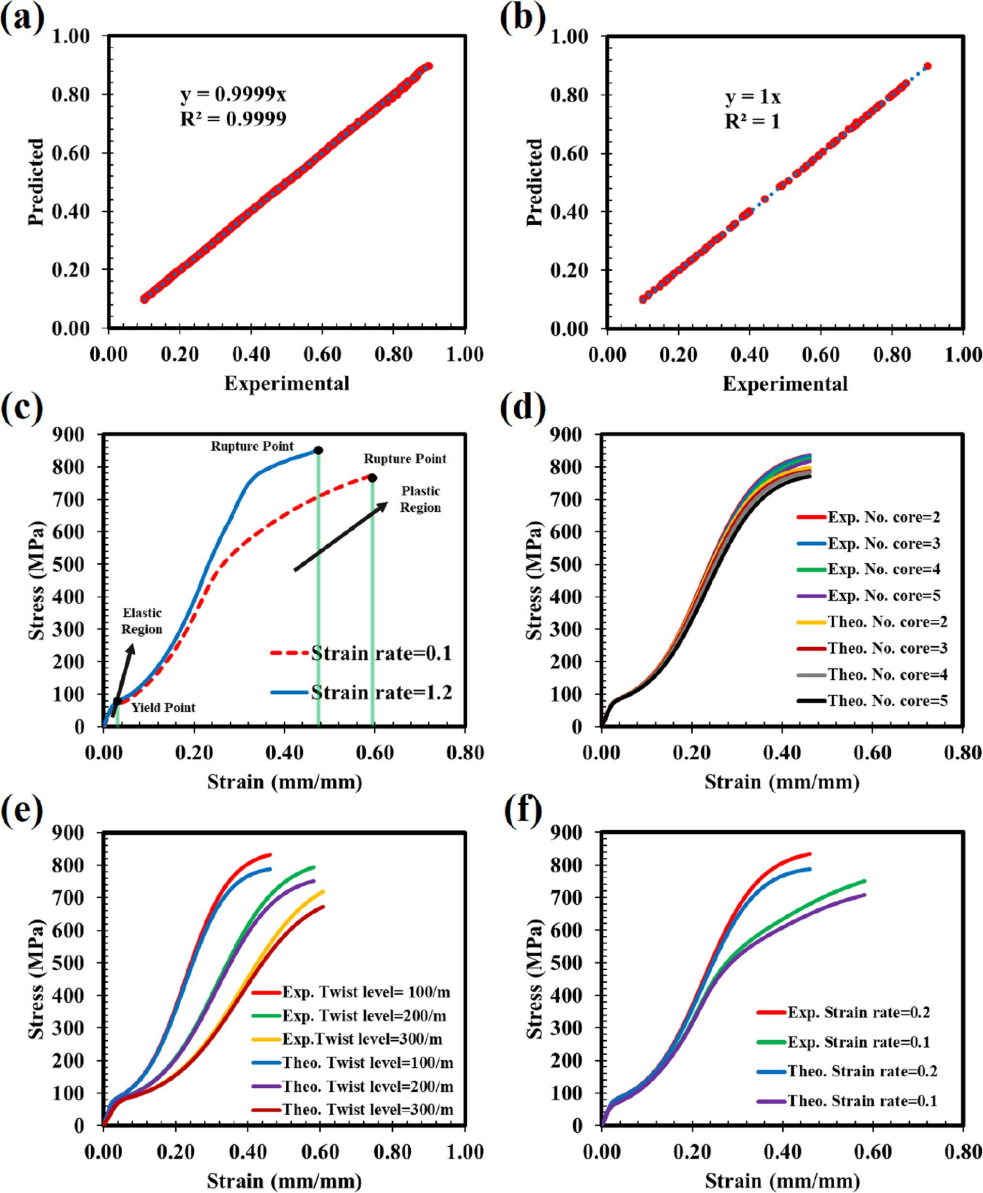
Performance of ANN model at (**a**) training step, (**b**) testing step, and stress-strain curve of (**c**) PA-monofilament at different rate of strain, (**d**) yarn made up of different numbers of PA-monofilament with twist level of 100 and strain rate of 1.2, (**e**) yarn made up of 3 PA-monofilaments with different twist levels and strain rate of 1.2, and (**f**) yarn made up of 3 PA-monofilaments with twist level of 100 and different strain rates.

Figure 6a-b illustrate the goodness of fit when an ANN model was trained and tested to map strain and strain rate to the force of core PA-monofilament. The results show a high correlation between normalized predicted and actual values (R² > 0.99), demonstrating the developed ANN model’s ability to predict force based on strain and strain rate with high precision. This model serves as an alternative to Hooke’s law for determining the acting force on monofilaments.

Figure 6c displays the tensile behavior of a PA-monofilament at two different strain rates (0.6 and 1.2). It is well-known that visco-elasto-plastic materials exhibit increased stress when subjected to higher strain rates. At higher strain rates, molecular chains have less time to reorient and relax, resulting in a stiffer response and higher stress levels for a given strain. Conversely, at lower strain rates, the chains have more time for coping with inserted stress, leading to a more compliant material with lower stress. It is important to note that the tensile behavior of polymeric materials such as PA is influenced by factors such as humidity, temperature, and strain rate^48,49^. While this work only considers strain rate, humidity and temperature could also be considered in future studies.

To validate the proposed model, a comparison was made between the predicted and experimental values, as shown in Fig. 6d-f. The samples were fabricated to investigate the effect of the number of monofilaments in the cross-section, twist level, and strain rate on the tensile behavior of multifilament yarn. It is evident that increasing the number of monofilaments from 2 to 5 results in a slight reduction in stress value due to the decreasing force component along the monofilament axis. This trend, is also observed in the theoretical curves with high correlation. The prediction domain extends from the elastic region to the plastic region, with a relatively high prediction error in the plastic region due to the friction of monofilaments near the rupture point in consistence with an another work^50^. Regarding the impact of twist level on tensile behavior, it was found that higher twist levels (from 100/m to 300/m) correspond to lower stress tolerated by the multifilament yarn. Increasing the twist level reduces the force component along the monofilament axis, such a trend also was evident in the theoretical curves. Similarly, when stretching continues until the rupture point, there is a low difference between theoretical and experimental values that can be attributed to the friction of monofilaments.

Lastly, it was found that increasing the strain rate from 0.6 to 1.2 results in multifilament yarn exhibiting similar behavior to a single monofilament. The higher the strain rate, the more stress is experienced by the multifilament yarn.

It should be mentioned that the twisting angle is dependent on the elongation of the monofilament in the axial direction and the compression of monofilaments in the lateral direction. Lateral deformation can result in a non-circular cross-section of the monofilaments. For nylon, a thermoplastic polymer, compressive strength is generally higher than tensile strength, a typical characteristic of many ductile polymers^51^. Under tensile stress, nylon’s polymer chains align with the applied load, stretching or necking before fracturing due to chain slippage or bond breaking. In contrast, compressive stress leads to plastic deformation, allowing the material to absorb more energy and resist failure, thanks to its semi-crystalline structure, which provides rigidity, and its ability to flow under compression. However, because the number of monofilaments is small and their diameters are limited, lateral deformation can be neglected in this case.

### Correlation between numerical and theoretical values

**Figure 7:**
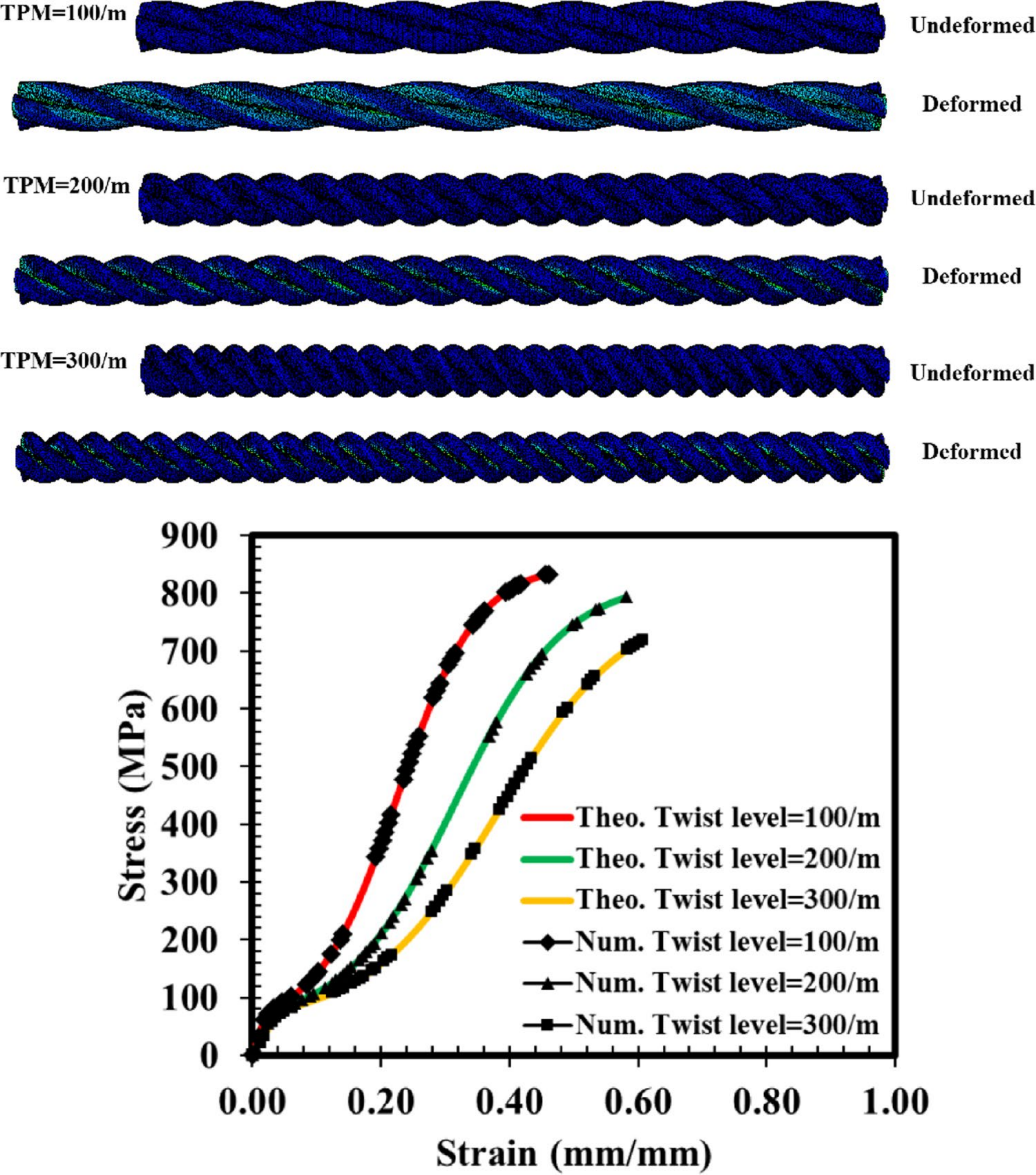
Numerical simulations and comparison between the numerical and theoretical values.

To evaluate the efficacy of the proposed modeling technique for multi-filament yarn, numerical simulations were conducted. Figure 7 presents the finite element analysis of three distinct multi-filament yarns with twist levels varying from 100/m to 300/m, along with a comparison between numerical and theoretical values.

The simulations involved creating and assembling 3D deformable helices using Abaqus Software. Material properties were defined using the complete tensile behavior at a strain rate of 1.2. The boundary conditions included one end of the structures being fixed while the other end was subjected to a strain of 0.6. Meshing was performed using tetrahedral elements with a mesh size of 0.1. The comparison results show a perfect correlation between the simulated and predicted values, that is attributed to the absence of friction in both theoretical and numerical analyses. In practice, factors such as necking in the plastic region and non-uniformity in monofilament properties introduce friction between the monofilament and the jaw near the rupture point, resulting in lower measured stress compared to theoretical values.

## Conclusion

In this study, a novel modeling approach is developed to predict the stress-strain behavior of the core region in multifilament yarns with core filament numbers ranging from 2 to 5, an aspect that has not been considered in previous researches. This method integrates geometric modeling and artificial neural network to predict the stress-strain curve in both the visco-elastic and visco-plastic regions. The experimental results demonstrate that the proposed model effectively interprets the number of cores, twisting level, and strain rate in the stress-strain curve of multifilament yarns. Numerical analysis indicates that in the absence of friction in the experimental stress-strain curve, there is a perfect agreement between experimental and theoretical values. The use of artificial neural networks, as opposed to traditional elastic models, offers significant advantages in modeling the mechanical behavior of textiles, particularly multifilament yarns. This method replaces conventional characterization techniques that rely on extracting indices such as Young’s modulus for linear relationships. Future research will focus on developing models for yarn structures with an infinite number of layers formed through the configuration of different core numbers using the same proposed method.

## Data Availability

The data that support the findings of this study are available on request from the corresponding authors.
